# Real-World Effectiveness of Belimumab in Patients with Active Lupus

**DOI:** 10.3390/jcm12247627

**Published:** 2023-12-11

**Authors:** Yuya Sumichika, Shuhei Yoshida, Eiji Suzuki, Kenji Saito, Haruki Matsumoto, Jumpei Temmoku, Yuya Fujita, Naoki Matsuoka, Tomoyuki Asano, Shuzo Sato, Kiyoshi Migita

**Affiliations:** 1 Department of Rheumatology, Fukushima Medical University School of Medicine, 1 Hikarigaoka, Fukushima 960-1295, Fukushima, Japan; ysumiti@fmu.ac.jp (Y.S.); s3xbck2p@fmu.ac.jp (K.S.); haruki91@fmu.ac.jp (H.M.); temmoku@fmu.ac.jp (J.T.); fujita31@fmu.ac.jp (Y.F.); naoki-11@fmu.ac.jp (N.M.); asanovic@fmu.ac.jp (T.A.); shuzo@fmu.ac.jp (S.S.); migita@fmu.ac.jp (K.M.); 2 Department of Rheumatology, Ohta Nishinouchi General Hospital Foundation, 2-5-20 Nishinouchi, Koriyama 963-8558, Fukushima, Japan; azsuzuki@ohta-hp.or.jp

**Keywords:** systemic lupus erythematosus, belimumab, intravenous cyclophosphamide, mycophenolate mofetil

## Abstract

This study evaluated the real-world effectiveness of belimumab (BLM) in the treatment of systemic lupus erythematosus (SLE) patients with moderate to high disease activity. This retrospective cohort study enrolled 129 Japanese patients with moderate to high SLE disease activity who received BLM between January 2013 and March 2023. The clinical outcomes, including the flare-free survival, SLE Disease Activity Index 2000 (SLEDAI-2K) score, and prednisone-equivalent dose, in the BLM and mycophenolate mofetil (MMF) treatment groups were compared before and after treatment. Safety data for BLM were collected. Additionally, we compared the effectiveness of BLM and intravenous cyclophosphamide (IV-CY) treatment using the stabilized inverse probability of treatment weighting (IPTW) method based on the propensity scores. This observational study enrolled 129 patients with moderate/severe SLE: 48 patients received belimumab, 45 received IV-CY, and 36 received MMF and prednisolone for remission induction therapy. The median follow-up for the BLM group was 17.0 months. Among them, 19 received BLM plus MMF. BLM significantly reduced the mean SLEDAI-2K (from mean baseline to 52 weeks: 49.2% reduction from 12.8 to 6.5) and prednisone daily dose (from mean baseline to 52 weeks: 21.9% reduction from 12.8 to 10.0 mg/day). The flare-free survival at 52 weeks was not significantly different between the BLM and MMF groups. There was no significant difference in the flare-free survival rates or reduction rates of the SLEDAI-2K between the patients treated with BLM and those treated with BLM plus MMF. In the propensity score-matched comparative analyses, there was no significant difference in the flare-free survival rates or an estimated decline in the SLEDAI-2K scores between the patients with lupus treated with BLM and IV-CY. BLM may be a promising alternative treatment option for lupus patients with moderate or high disease activity who do not respond to conventional treatments.

## 1. Introduction

Systemic lupus erythematosus (SLE) is a chronic autoimmune disease characterized by a loss of self-tolerance and the aberrant formation of anti-double-stranded (ds) DNA antibodies or anti-Sm antibodies, leading to multiple organ involvement [1,2]. The current treatment options for SLE include glucocorticoids (GCs), hydroxychloroquine (HCQ), and the immunomodulatory agents rituximab and cyclophosphamide (CY) [2]. Immunosuppressive therapy for SLE includes azathioprine, cyclosporine A, tacrolimus, and mycophenolate mofetil (MMF), which are used to treat patients with lupus with moderate to high disease activity [2,3]. Furthermore, intravenous cyclophosphamide (IV-CY) is an established therapeutic option in lupus patients with high disease activity [4]. Belimumab (BLM) is a human immunoglobulin G1λ monoclonal antibody that antagonizes B cell activation factor (BAFF), thus preventing the interaction of BAFF with its three receptors and indirectly decreasing the B cell survival and production of autoantibodies [5]. Clinical trial data suggest a promising role for targeting BAFF in treating active SLE [6,7]. BLM has been approved for the treatment of active SLE via both intravenous (IV) and subcutaneous (SC) injections in Japan. Recently, two international phase III trials, i.e., BLISS-52 and BLISS-76, revealed similar safety profiles and significantly higher response rates, as measured by the Systemic Lupus Erythematous Responder Index (SRI) [8,9]. Additionally, in the BLISS-LN trial involving patients with active lupus nephritis, more patients who received belimumab plus standard therapy had a primary efficacy renal response than those who received standard therapy alone [10]. Overall, BLM is safe and well-tolerated [11]. Recent observational studies have also demonstrated the efficacy of BLM in reducing disease activity and SLE flares, enabling patients to taper their glucocorticoid use and limit organ damage caused by SLE [12]. These studies suggest that belimumab could be an effective therapeutic option to treat active SLE, allowing the sparing of glucocorticoids; however, the effectiveness of BLM in treating moderate to severe SLE has not been fully explored in a real-world clinical setting. Therefore, the effectiveness of belimumab in treating SLE needs to be determined. This novel study aimed to evaluate the clinical efficacy of belimumab in comparison to other treatment options in the Japanese population. We compared the effectiveness of BLM with or without MMF and the induction of remission with BLM versus IV-CY in the treatment of SLE moderate to high disease activity.

## 2. Materials and Methods

### 2.1. Study Design

The cohort consisted of patients with SLE treated at the Department of Rheumatology of Fukushima Medical University Hospital. Between January 2013 and March 2023, 253 patients with SLE were treated. Patients were included in this study if they fulfilled the revised criteria for SLE set by the 1997 American College of Rheumatology [13]. Among these patients, 129 were enrolled in the study who met the following criteria: had a documented SLE diagnosis, were ≥18 years of age at study entry, and continued BLM (10 mg/kg intravenous or 200 mg subcutaneous injection) or IV-CY (500 mg per square meter of body surface area) plus standard therapy for at least two infusions at the time of the study/or received MMF and glucocorticoid induction remission therapy enrolment. Only patients with a baseline SLEDAI-2K score of ≥6 were included. Stable doses of hydroxychloroquine, tacrolimus, cyclosporine A, and mycophenolate mofetil were concomitantly administered. Patients were excluded from the study if they were initiated with BLM at the other hospitals if they did not have a medical history prior to BLM initiation or if they had received anifrolumab or IV-CY within 2 years before or simultaneously. “Exposure” was defined as the period from the initiation of BLM, IV-CY, or MMF treatment until the discontinuation of the treatment or the patient’s transfer to another hospital, death, or the end of the study period, whichever occurred first. The study was conducted in accordance with the Declaration of Helsinki and approved by the institutional review boards of Fukushima Medical University (No. 2020-110). Owing to the retrospective study design, an opt-out strategy was chosen for the participants, and those who declined to provide informed consent were excluded.

### 2.2. Data Collection

The collected data included complete basic patient information, clinical data, SLE disease activity assessment, and an assessment of BLM efficacy after 26 and 52 weeks of treatment. The basic information included age at disease onset, sex, cause of medication, and course of illness. Blood and urine laboratory tests, medical histories, and the clinical findings of patients with SLE were collected by reviewing electronic medical records. The immunological tests included the detection of anti-double-stranded DNA ((anti-dsDNA) antibody analysis) and the measurement of complement (C) 3 and C4 levels. SLE disease activity was assessed using the Systemic Lupus Erythematosus Disease Activity Index 2000 (SLEDAI-2K) [14].

SLE disease activity was categorized based on the SLEDAI-2K scores as follows: no activity (SLEDAI-2K = 0), mild activity (SLEDAI-2K = 1–5), moderate activity (SLEDAI-2K = 6–10), high activity (SLEDAI-2K = 11–19), or very high activity (SLEDAI-2K > 20) [15]. A flare was defined as an elevated SLEDAI-2K of 3 or greater, an increase in prednisolone dose of 5 mg or greater, or BILAG B or greater [16], and a clinical condition that required the addition of immunosuppressive agents.

### 2.3. Statistical Analysis

The continuous variables were summarized as the means ± standard deviations or the medians with interquartile ranges. The categorical variables were reported as frequencies and percentages. The differences between the two groups were analyzed using the Student’s *t*-test for normally distributed continuous variables, the Mann–Whitney U-test for non-normally distributed continuous variables, and Fisher’s exact test for categorical variables. The statistical significance for all the tests was defined as a two-tailed *p*-value of <0.05. The stabilized inverse probability of treatment weighting (IPTW) based on the propensity scores was used to adjust for the differences in the covariates between the two groups [17]. We estimated the propensity score using a multivariable logistic regression model, including the following prespecified confounding factors: age, sex, lupus nephritis, neuropsychiatric SLE, SLEDAI-2K score, tacrolimus or cyclosporine A use, mycophenolate mofetil use, and prednisolone (PSL) dose. The factors had no missing data. The balance in the baseline clinical characteristics was assessed between the two groups before and after the propensity score weighting using the absolute standardized mean differences, with values of <0.1 indicating a good balance [18]. A generalized linear model was used to estimate the treatment effect to calculate the SLEDAI-2K decline estimates for BLM using IV-CY as a reference at 26 and 52 weeks. The time to flare in the BLM-treated and IV-CY-treated groups was estimated using Kaplan–Meier analysis, and log-rank tests were used to compare the cumulative flare-free proportion between the patient groups. The statistical analyses were performed using SPSS Statistics software (version 29.0; IBM Corp., Armonk, NY, USA) and R ver. 4.3.1 (R Foundation for Statistical Computing, Vienna, Austria, http://www.R-project.org/, accessed on 26 July 2023).

## 3. Results

### 3.1. Patients’ Baseline Characteristics

**Figure 1:**
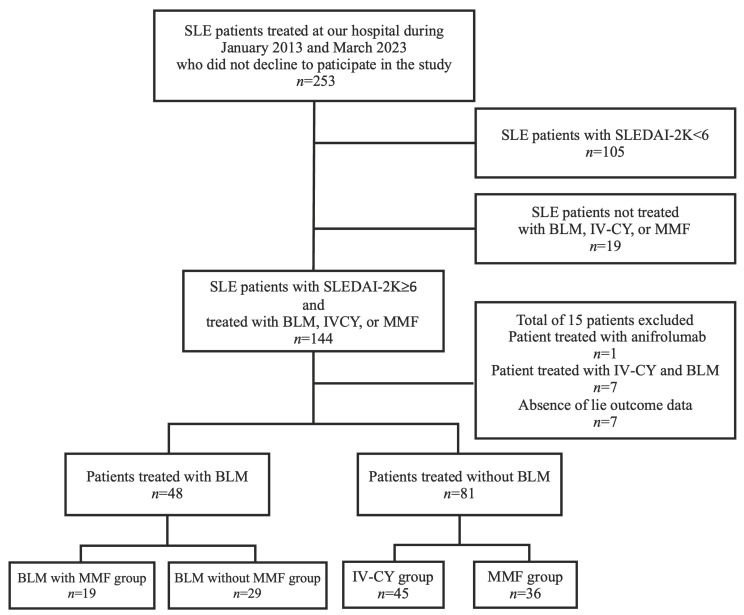
Flow chart showing patient enrollment. Among 253 patients with SLE who were initially treated with BLM, MMF, or IV-CY due to moderate to high SLE activity (SLEDAI-2K ≥ 6) at our institution between January 2013 and March 2023, 129 were enrolled in this study. SLE: systemic lupus erythematosus, SLEDAI-2K: SLE Disease Activity Index 2000, IV-CY: intravenous cyclophosphamide, BLM: belimumab, MMF: mycophenolate mofetil.

Among the 253 patients with SLE who began treatment at our institution between January 2013 and March 2023, 129 were enrolled in the study (Figure 1). The baseline demographic and clinical characteristics of the BLM and control groups are indicated in Table 1. Most BLM-treated patients (68.8%; 33 of 48) received subcutaneous belimumab intravenous belimumab (200 mg, 2 weeks interval) compared with 31.3% (15 of 48) who received intravenous belimumab (10 mg/kg, 4 weeks interval). The mean ± standard deviation age of the patients was 40.0 ± 11.5 years, with 43 (89.6%) patients being female; the median (interquartile range) disease duration was 10.0 (4.2–20.0) years; and the median (interquartile range) follow-up duration was 17.0 (8.3–27.8) months. In total, 31 patients (64.5%) had lupus nephritis. The SLEDAI-2K, an SLE disease activity measure, was 13.5 (10.0–16.0), and all the patients had moderate to high disease activity. All the patients were treated with corticosteroids, and 31 patients (64.6%) were under hydroxychloroquine treatment before starting with belimumab. Mycophenolate mofetil (*n* = 19, 39.6%; mean doses 1460 ± 690 mg/day) was the most commonly prescribed immunosuppressant, followed by calcineurin inhibitors, such as cyclosporine and tacrolimus (*n* = 18, 37.5%). During the period of treatment with BLM, adverse events were observed in 5 patients (1: urinary tract infection, 1: disseminated herpes zoster, 2: skin rash, and 1: depression). One patient with depression resulted in the drug discontinuation. The control group had significantly shorter disease duration, more neuropsychiatric, cardiorespiratory, lupus nephritis, and hematological symptoms, higher SLEDAI-2K, less HCQ, TAC, and CyA use, and a higher PSL dose than the BLM group.

### 3.2. Changes in Clinical Parameters after Treatment with BLM

**Figure 2:**
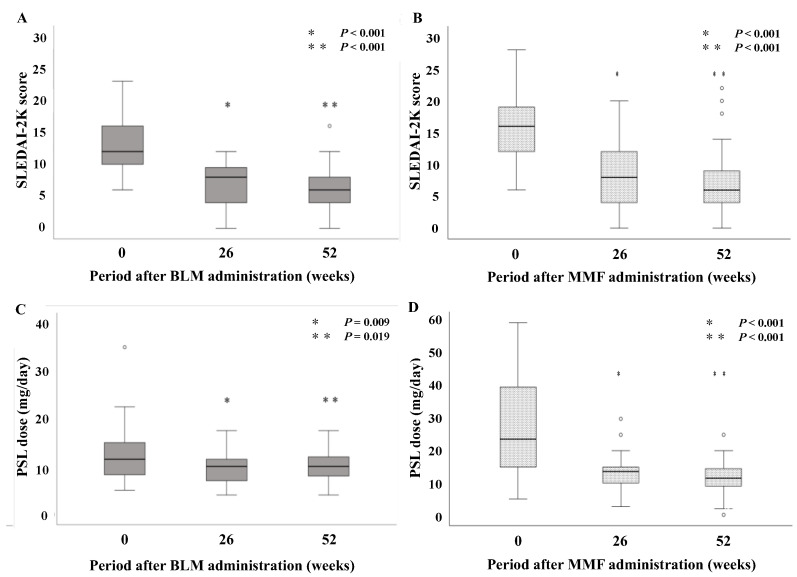
Change from baseline in the SLEDAI-2K score and prednisolone dose at 26 weeks or 52 weeks from the start of BLM therapy and MMF therapy. Data are presented as box plots and whiskers. SLEDAI-2K scores were significantly reduced at 26 or 52 weeks from the start of BLM (**A**) or MMF (**B**) therapy compared to baseline data. PSL doses also significantly decreased at each time point from the start of BLM (**C**) or MMF (**D**) therapy compared to baseline data. SLEDAI-2K: SLE Disease Activity Index 2000, BLM: belimumab, MMF: mycophenolate mofetil, PSL: prednisolone.

**Figure 3:**
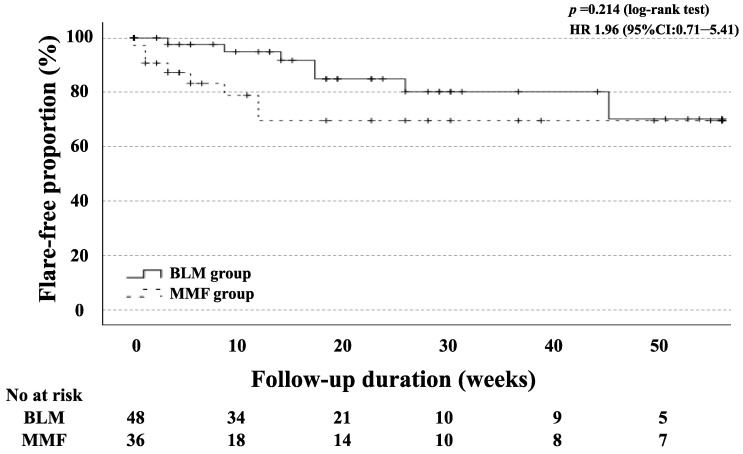
Kaplan–Meier curves showing the flare-free survival in patients treated with BLM (*n* = 48) and MMF (*n* = 36). No significant differences were observed between BLM-treated and MMF-treated groups. The starting point (0 years) was the date on which the observations began.

In our study, the mean follow-up time for the BLM group was 17.0 (8.3–27.8) months. The changes in their clinical parameters before and after treatment with BLM or MMF + PSL are shown in Figure 2. The mean SLEDAI-2K score for the BLM group was 12.8 at baseline, 7.0 at 26 weeks, and 6.5 at 52 weeks. After 26 weeks and 52 weeks of treatment, the SLEDAI-2K scores significantly decreased compared to those at baseline (Figure 2A; both *p* < 0.001). The mean SLEDAI-2K score for the MMF group was 15.8 at baseline, 8.7 at 26 weeks, and 7.5 at 52 weeks. After 26 and 52 weeks of treatment, as in the BLM group, the SLEDAI-2K scores were significantly lower than at baseline, respectively (Figure 2B; both *p* < 0.001). The mean prednisolone (PSL) dose for the BLM group was 12.8 mg/day at baseline, 9.8 mg/day after 26 weeks, and 10.0 mg/day after 52 weeks. Similarly, after 26 weeks and 52 weeks of treatment, the doses of PSL were significantly reduced compared with those at baseline (Figure 2C; *p* = 0.009 and *p* = 0.019). The mean prednisolone (PSL) dose for the MMF group was 28.0 mg/day at baseline, 13.8 mg/day after 26 weeks, and 11.6 mg/day after 52 weeks. As in the BLM group, after 26 weeks and 52 weeks of treatment, the doses of PSL were significantly reduced compared with those at baseline (Figure 2D; both *p* < 0.001). The flare-free survival at 52 weeks after remission induction therapy was not significantly different between the two groups (Figure 3).

### 3.3. Changes in Clinical Parameters after Treatment with Belimumab with or without Concomitant MMF Use

**Figure 4:**
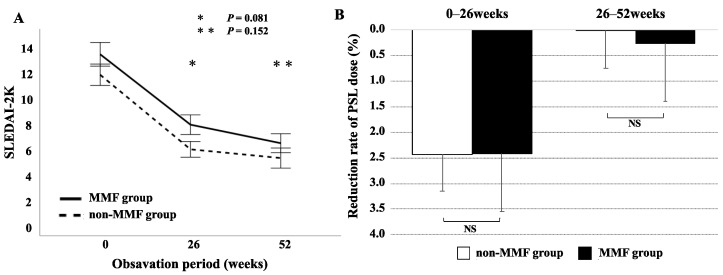
Reduction rates of SLEDAI-2K score (**A**) and PSL dose (**B**) in lupus patients treated with BLM or BLM plus MMF. Reduction rates of SLEDAI-2K score or PSL dose were calculated according to the baseline SLEDAI-2K score or PSL dose (% of reduced PSL dose/baseline PSL dose or % of SLEDAI-2K score/baseline SLEDAI-2K score). There were no significant differences in the reduction rates of SLEDAI-2K score or PSL dose between lupus patients treated with BLM and BLM plus MMF treatment at each time point (26 weeks and 52 weeks). SLEDAI-2K: SLE Disease Activity Index 2000, MMF: mycophenolate mofetil, PSL: prednisolone, NS: not significant.

**Figure 5:**
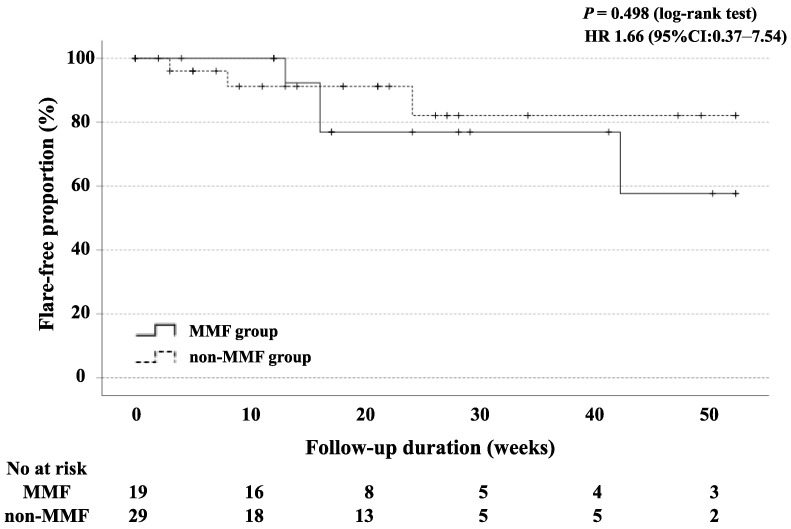
Kaplan–Meier curves showing the cumulative flare-free survival rates in lupus patients who were treated with BLM (*n* = 29) and BLM and MMF combination therapy (*n* = 19). No significant differences were observed between MMF and non-MMF groups. MMF: mycophenolate mofetil, HR: hazard ratio.

Among 48 patients initiated with BLM, 19 were classified in the MMF group (BLM plus MMF) and 29 in the non-MMF group with BLM alone. We tried to compare the clinical course between these SLE patients treated with BLM and co-treated with and without the use of MMF. The baseline demographics and clinical characteristics of both groups are shown in Table 2. With the exception of lupus nephritis, there was no significant difference in the baseline demographic data between the MMF and non-MMF groups. The changes in the SLEDAI-2K and PSL doses after BLM administration in both groups are shown in Figure 4. Both groups significantly reduced the SLEDAI-2K and PSL doses at 56 weeks compared to the baseline; however, the difference was not statistically significant between the two groups. Similarly, there was no significant difference in the flare-free survival rates between the MMF and non-MMF groups (Figure 5). There was no significant difference in renal involvement, including the changes in eGFR or proteinuria between the patients treated with BLM with or without the concomitant use of MMF.

### 3.4. Comparisons of Clinical Responses between Patients Treated with Belimumab and IV-CY

**Figure 6:**
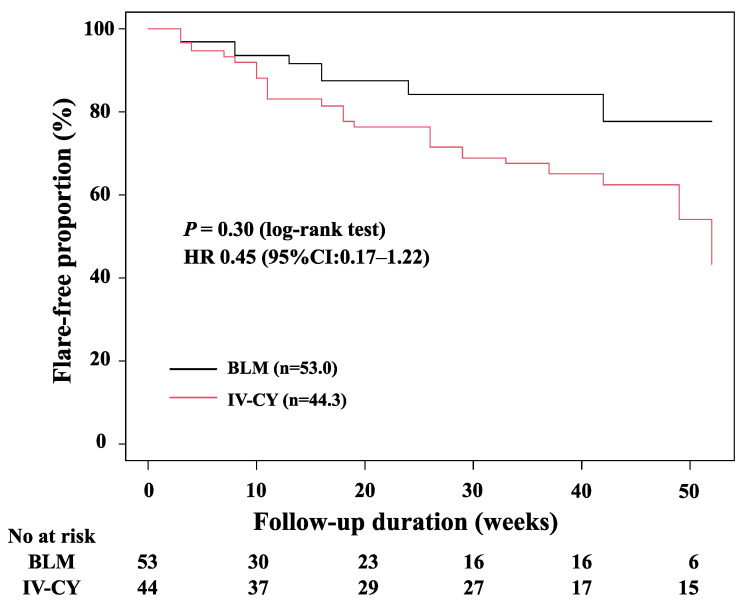
The relapse-free survival rate in the BLM (*n* = 53.0) and IV-CY groups (*n* = 44.3) after adjusting for propensity score-based stabilized IPTW. No significant differences were observed in the cumulative flare-free survival rates between lupus patients treated with BLM and IV-CY. BLM: belimumab, IV-CY: intravenous cyclophosphamide, HR: hazard ratio, IPTW: inverse probability of treatment weighting.

The background characteristics of the patients in the IV-CY-treated and BLM-treated groups before and after the stabilized inverse probability of treatment weighting (IPTW) are summarized in Table 3. A comparison of the two groups before the stabilized IPTW showed a significantly shorter disease duration, a longer observation period, a higher SLEDAI-2K, a higher PSL dose, a lower rate of concomitant immunosuppressive drugs, and more NPSLE, mucocutaneous, and cardiorespiratory symptoms in the IV-CY group. After the stabilized IPTW, the SLEDAI-2K, PSL dose, immunosuppressant administration, and phenotypes associated with poor prognoses, such as NPSLE and lupus nephritis, were balanced in both groups. After the stabilized IPTW, the bias in the distribution of the propensity scores for both groups was adjusted (Supplementary Figure S1). A comparison of the flare-free survival between the two groups is shown in Figure 6. Although the patients in the IV-CY group appeared to have slightly higher rates of flares than those in the BLM group, the cumulative incidences of the flares were almost the same between the two groups after adjusting for the stabilized IPTW based on the propensity scores. Estimates of the decrease in the SLEDAI-2K in the BLM group relative to the IV-CY group after the IPTW were calculated (Table 4). The estimated decrease in the SLEDAI-2K was −1.05 (*p* = 0.76, 95%CI: −7.66–5.57) at 26 weeks and −0.99 (*p* = 0.71, 95%CI: −6.18–4.19) at 56 weeks, indicating no significant difference in the estimated decrease in the SLEDAI-2K scores between BLM and IV-CY after IPTW stabilization.

## 4. Discussion

Most patients with SLE experience symptom exacerbations or flares during the disease course, which may cause long-term organ damage [19]. Therefore, preventing flares is an important issue in the management of patients with lupus [20]. The prolonged use of GC may cause irreversible organ damage; therefore, it was listed as the first drug to be withdrawn during the maintenance stage of lupus therapy [21]. Conventional immunomodulatory agents, such as mycophenolate mofetil (MMF), azathioprine, cyclosporine A, and tacrolimus, are widely used in the management of active SLE [22]. However, these drugs are nonspecific and their long-term use may increase the risk of organ damage [23]. Recent clinical trial data indicated a significant benefit of belimumab in the treatment of lupus patients [6,7]. These data suggest that the addition of belimumab to standard immunosuppressive treatments may be effective in the treatment of active SLE. However, only a few real-world studies have reported the efficacy of belimumab [24]. We analyzed the clinical effectiveness of belimumab treatment in patients with moderate to high SLE disease activity.

In this study, patients with moderate to high SLE disease activity who were initiated on belimumab were enrolled, and their clinical outcomes, including flare-free survival rates and changes in the SLEDAI-2K score, were assessed. In our study, the flare-free survival rate was 70.2% at 56 weeks in the belimumab-treated lupus patients with moderate to high SLE disease activity. In our study, the SLEDAI-2K significantly decreased after 26 to 52 weeks from the initiation of BLM treatment compared with those at baseline. The most frequent adverse event associated with BLM was minor infection. However, in addition to the use of BLM, the disease activity, prednisolone dose, and concomitant immunosuppressive treatments may be involved in the observed adverse events. Our data suggest the real-world effectiveness of BLM in controlling moderate to high SLE disease activity, which is consistent with the findings of previous studies [24].

Mycophenolate mofetil (MMF) is commonly used for the treatment of SLE [25]. Previous studies have suggested that BLM may provide additional benefits in patients with the combined use of MMF in clinical improvements, including a reduction in prednisolone doses and SLEDAI-2K scores [26]. However, in contrast to these studies, there was no significant difference in the flare-free survival rates, reduction rates of the SLEDAI-2K score, and reduction rates of the prednisolone dose between the lupus patients treated with BLM and those treated with BLM plus MMF. The disease durations of the enrolled patients were longer, which may contribute to the differential results in our study compared with those of previous studies [6,8,26]. Additionally, the BLM + MMF group included more lupus nephritis associated with a poor prognosis than the non-MMF group [27]. Furthermore, the number of participants in our study was limited; thus, further large-scale studies are required to address these important issues.

The main reasons for the initiation of immunosuppressive treatments were disease progression, inability to control SLE activity, and inability to reduce the prednisolone dose using the current treatment regimen [28]. The effectiveness of intravenous cyclophosphamide (IV-CY) in the treatment of moderate to severe SLE has been consistently demonstrated [29]. However, IV-CY still leads to short and long-term AEs in terms of fertility, teratogenicity, and carcinogenicity, which have been concerns, in addition to the risk of infection [30].

We evaluated the effectiveness of IV-CY versus BLM in the treatment of patients with moderate to high SLE disease activity isolated from the same SLE dataset. As significant differences were observed in the baseline clinical characteristics between the patients receiving BLM and those receiving IV-CY, we performed a comparative analysis using propensity score weighting to correct the selection bias. Although a few variables could not be adjusted for in the IV-CY and BLM groups and even in the propensity score matching groups, our results suggest that there were no significant differences in the cumulative flare-free survival rates between the groups. The reduction rates in the SLEDAI-2K scores were similar between the two groups. Although there were some differences in the demographic background data, our results suggest that BLM may be as effective as an IV-CY in patients with moderate to high SLE disease activity.

Our study had several limitations. The study design is a retrospective observational design and does not clearly demonstrate the effectiveness of BLM. This study had a small sample size and enrolled a limited number of patients with SLE. Our study was conducted at only two institutions. Therefore, the results should be validated in a multicenter study. Although we used propensity score-based IPTW to minimize the influence of confounding factors, there may have been residual differences, and not all the variables were adjusted for between the IV-CY and BLM groups. The drug administration and glucocorticoid dose reduction were performed conventionally and not according to the standard protocols. The overall SLE activity was evaluated according to the SLEDAI-2K score and was unable to evaluate the activity of each organ manifestation due to the small sample size. Furthermore, all the conclusions are limited because of the large number of censoring in this study at the 52-week follow-up period from remission induction to 52 weeks. Due to the relatively short approval time for clinical application, only a limited number of patients with active lupus receiving BLM were enrolled in this study. Therefore, our findings may not apply to all patients with active SLE.

## 5. Conclusions

In this real-world setting, our study demonstrated that BLM for the treatment of moderate to severe SLE is associated with improvements in the SLEDAI-2K scores and reductions in the daily prednisolone-equivalent dose. BLM may be a promising alternative treatment option for lupus patients with moderate or high disease activity who do not respond to conventional treatments.

## Figures and Tables

**Table 1:** Demographic and clinical characteristics of the lupus patients treated with BLM at baseline.

Characteristic	BLM (*n* = 48)	Control (MMF) (*n* = 36)	*p* (Value)
Female, *n* (%)	43 (89.6)	28 (77.8)	0.14
Age at enrollment, † years	41.0 (31.0–47.0)	41.0 (29.0–49.3)	0.93
Subcutaneous injection, *n* (%)	33 (68.8)		
Intravenous injection, *n* (%)	15 (31.3)		
- dose, mg/month	520 (450–640)		
Disease duration, † years	10.0 (4.2–20.0)	3.5 (0–17.5)	0.03 *
Observation period, † month	17.0 (8.3–27.8)	9.0 (1.25–35.5)	0.27
Mucocutaneous, *n* (%)	25 (53.1)	15 (41.7)	0.33
Neuropsychiatric, *n* (%)	2 (4.2)	7 (19.4)	0.03 *
Musculoskeletal, *n* (%)	17 (35.4)	15 (41.7)	0.56
Cardiorespiratory, *n* (%)	2 (4.2)	9 (25.0)	0.01 *
Gastrointestinal, *n* (%)	6 (12.5)	5 (13.9)	0.85
Lupus nephritis, *n* (%)	31 (64.5)	32 (88.9)	0.01 *
Haematological, *n* (%)	10 (20.8)	15 (41.7)	0.04 *
SLEDAI-2K, †	13.5 (10.0–16.0)	16.0 (12.0–19.5)	0.01 *
Serum C3, † g/L	71.5 (56.3–94.0)	62.5 (43.8–88.3)	0.07
Serum C4, † g/L	13.9 (8.0–20.0)	10.5 (5.3–17.8)	0.17
Serum creatinine, † mg/dL	0.67 (0.58–0.87)	0.85 (0.66–1.06)	0.03 *
eGFR, † mL/min/1.73 m^2^	81.2 (61.6–95.0)	66.5 (50.0–88.3)	0.07
CRP, † mg/dL	0.09 (0.05–0.27)	0.22 (0.07–0.48)	0.08
Anti-dsDNA titer, † IU/mL	18.0 (1.4–48.7)	20 (2.5–127.0)	0.23
Anti-dsDNA positive, *n* (%)	45 (93.7)	35 (97.2)	0.46
Urinary protein, † g/g Cre	0.47 (0.09–1.47)	2.05 (0.72–2.92)	0.01 *
HCQ use, *n* (%)	31 (64.6)	13 (36.1)	0.01 *
MMF use, *n* (%)	19 (39.5)	36 (100)	0.001 *
TAC or CyA use, *n* (%)	18 (37.5)	6 (16.7)	0.04 *
PSL dose, † mg/day	11.5 (8.3–15.0)	23.8 (15.0–40.0)	0.001 *

† Values are presented as medians [IQR], and *n* (%) was used for the categorical variables. * means there is a significant difference at *p* < 0.05. SLE: systemic lupus erythematosus, BLM: belimumab, SLEDAI-2K: SLE Disease Activity Index 2000, CRP: C-reactive protein, DNA: deoxyribonucleic acid, HCQ: hydroxychloroquine, MMF: mycophenolate mofetil, TAC: tacrolimus, CyA: cyclosporine A, PSL: prednisolone.

**Table 2:** Comparisons of clinical features between MMF group and non-MMF group.

Characteristics	All Patients		
	MMF (*n* = 19)	Non-MMF (*n* = 29)	*p* (Value)
Female, *n* (%)	16 (84.0)	27 (93.0)	0.33
Age at enrollment, † years	34.0 (32.0–46.0)	42.0 (32.3–47.8)	0.17
Disease duration, † years	9.0 (5.0–17.0)	12.0 (4.0–20.5)	0.49
Observation period, † month	17.0 (12.0–39.0)	18.0 (6.0–27.0)	0.90
Mucocutaneous, *n* (%)	9 (47.3)	16 (55.2)	0.60
Neuropsychiatric, *n* (%)	1 (5.3)	1 (3.4)	0.76
Musculoskeletal, *n* (%)	6 (31.6)	11 (37.9)	0.65
Cardiorespiratory, *n* (%)	1 (5.3)	1 (3.4)	0.76
Gastrointestinal, *n* (%)	2 (10.5)	4 (13.8)	0.74
Lupus nephritis, *n* (%)	16 (84.2)	15 (51.7)	0.02 *
Haematological, *n* (%)	2 (10.5)	8 (27.6)	0.16
SLEDAI-2K, †	14.0 (10.0–16.0)	12.0 (8.0–16.0)	0.22
Serum creatinine, † mg/dL	0.78 (0.62–0.94)	0.65 (0.58–0.80)	0.20
eGFR, † mL/min/1.73 m^2^	74.0 (60.0–97.0)	82.4 (68.0–95.0)	0.52
Serum C3, † g/L	72.0 (61.0–91.0)	70.0 (52.5–104.0)	0.76
Serum C4, † g/L	15.0 (8.0–20.0)	13.8 (7.3–21.0)	0.66
CRP, † mg/dL	0.13 (0.05–0.43)	0.06 (0.35–0.19)	0.07
Anti-dsDNA titer, † U/mL	27.7 (1.17–73.0)	12.0 (1.5–43.6)	0.35
Urinary protein, † g/g Cre	1.00 (0.36–2.02)	0.34 (0.07–1.28)	0.42
HCQ use, *n* (%)	13 (68.4)	18 (62.1)	0.76
TAC or CyA use, *n* (%)	4 (21.1)	14 (48.3)	0.13
PSL dose, † mg/day	12.5 (10.0–16.0)	11.0 (8.0–15.0)	0.61

† Values are presented as medians [IQR], and *n* (%) was used for the categorical variables. * means there is a significant difference at *p* < 0.05. SLE: systemic lupus erythematosus, MMF: mycophenolate mofetil, SLEDAI-2K: SLE Disease Activity Index 2000, CRP: C-reactive protein, DNA: deoxyribonucleic acid, HCQ: hydroxychloroquine, TAC: tacrolimus, CyA: cyclosporine A, PSL: prednisolone.

**Table 3:** Comparisons of clinical features between IV-CY group and BLM group.

Characteristics	All Patients			Stabilized IPTW		
	IV-CY (*n* = 45)	BLM (*n* = 48)	*p*-Value	IV-CY (*n* = 44.3)	BLM (*n* = 53.0)	*p*-Value
Female, *n* (%)	39 (86.7)	43 (89.6)	0.75	39.0 (88.0)	47.5 (89.5)	0.85
Age at BLM or IV-CY introduction, † years	34.0 (26.0–46.0)	41 (32.0–47.3)	0.11	43.2 (19.0–70.0)	42.3 (20.0–69.0)	0.90
Number of injections, † times	6 (3–7.5)			6 (3–7.5)		
Disease duration, † years	1 (0–5.0)	10.0 (4.2–20.0)	0.001 *	0.0 (0.0–19.0)	10.9 (0.0–32.0)	<0.001 *
Observation period, † month	33 (11.5–67.5)	17.0 (8.3–27.8)	0.003 *	39.8 (0.0–126.0)	12.0 (0.0–57.0)	0.002 *
SLEDAI-2K †	16.0 (8.0–22.0)	12.0 (10.0–16.0)	0.04 *	8.5 (6.0–38.0)	16.0 (6.0–23.0)	0.20
Serum C3, † g/L	65 (41.5–91.2)	71.5 (56.3–94.0)	0.12	56.6 (22.0–121.0)	61.0 (33.0–146.0)	0.22
Serum C4, † g/L	10.0 (5.2–18.5)	13.9 (8.0–20.0)	0.09	7.0 (1.0–32.0)	9.2 (1.0–51.0)	0.93
Anti-dsDNA titer, † U/mL	24.0 (4.0–66.0)	18.0 (1.4–48.7)	0.25	24.1 (2.0–68.0)	17.6 (1.2–44.6)	0.07
HCQ use, *n* (%)	10 (22.2)	31 (64.6)	0.001 *	7.8 (17.7)	40.8 (77.0)	<0.001 *
Concomitant MMF use, *n* (%)	1 (2.2)	19 (39.6)	<0.001 *	1.8 (4.0)	8.3 (15.7)	0.16
Concomitant TAC or CyA use, *n* (%)	5 (11.1)	18 (37.5)	0.004 *	9.3 (22.9)	11.9 (24.9)	0.06
PSL dose, † mg/day	40.0 (10.0–55.0)	11.5 (5.0–22.5)	0.04 *	20.0 (10.0–35.0)	14.5 (5.0–22.5)	0.64
Mucocutaneous, *n* (%)	11 (24.4)	25 (53.1)	0.01 *	8.4 (19.0)	33.4 (63.0)	0.003 *
Neuropsychiatric, *n* (%)	15 (33.3)	2 (4.2)	<0.001 *	8.9 (20.2)	16.5 (31.1)	0.58
Musculoskeletal, *n* (%)	10 (22.2)	17 (35.4)	0.16	6.4 (14.4)	11.8 (22.2)	0.41
Cardiorespiratory, *n* (%)	9 (20.0)	2 (4.2)	0.02 *	8.5 (19.1)	1.8 (3.3)	0.03 *
Gastrointestinal, *n* (%)	9 (20.0)	6 (12.5)	0.33	7.6 (17.2)	4.8 (9.1)	0.30
Lupus nephritis, *n* (%)	27 (60.0)	31 (64.6)	0.67	22.9 (51.6)	26.7 (50.4)	0.95
Haematological, *n* (%)	14 (31.1)	10 (20.8)	0.26	12.2 (27.5)	7.9 (14.9)	0.23

† Values are presented as medians [IQR], and *n* (%) was used for the categorical variables. * means there is a significant difference at *p* < 0.05. IV-CY: intravenous cyclophosphamide, BLM: belimumab, IPTW: inverse probability of treatment weighting, DNA: deoxyribonucleic acid, SLEDAI-2K: SLE Disease Activity Index 2000, MMF: mycophenolic acid mofetil, TAC: tacrolimus, CyA: cyclosporine A.

**Table 4:** SLEDAI-2K decline estimates for BLM using IV-CY as a reference after adjusting for propensity score-based stabilized IPTW.

	Estimated Decrease in SLEDAI-2K Scores	95%CI	*p*-Value
SLEDAI-2K score decrease (26 w)	−1.05	−7.66~5.57	0.76
SLEDAI-2K score decrease (52 w)	−0.99	−6.18~4.19	0.71

BLM: belimumab, IV-CY: intravenous cyclophosphamide, IPTW: inverse probability of treatment weighting, CI: confidence interval, SLEDAI-2K: SLE Disease Activity Index-2000.

## Data Availability

The raw data supporting the conclusions of this article will be made available by the authors, without undue reservation.

## Supplementary Materials

The following supporting information can be downloaded at: https://www.mdpi.com/article/10.3390/jcm12247627/s1, Figure S1: Kernel density estimates of the propensity score before and after IPTW adjustment. Probability densities of the distribution of propensity scores before and after stabilized IPTW for the BLM and IV-CY groups are shown. The propensity scores of the two groups were quite different before stabilized IPTW adjustment; however, after adjustment, the bias in the propensity scores was adjusted. BLM: belimumab, IV-CY: intravenous cyclophosphamide, IPTW: inverse probability of treatment weighting.

## Abbreviations

BAFF	B cell activation factor
BLM	belimumab
DNA	deoxyribonucleic acid
GCs	glucocorticoids
HCQ	hydroxychloroquine
IV-CY	intravenous cyclophosphamide
IPTW	inverse probability of treatment weighting
MMF	mycophenolate mofetil
PSL	prednisolone
SLE	systemic lupus erythematosus
SLEDAI-2K	Systemic Lupus Erythematosus Disease Activity Index 2000
SRI	Systemic Lupus Erythematous Responder Index
