# Supplementary figures and images for: Real-World Effectiveness of Belimumab in Patients with Active Lupus
Source: J Clin Med. 2023 Dec 11;12(24):7627. doi: 10.3390/jcm12247627 (PMC10743796; doi:10.3390/jcm12247627)

Supplementary  
figure S1

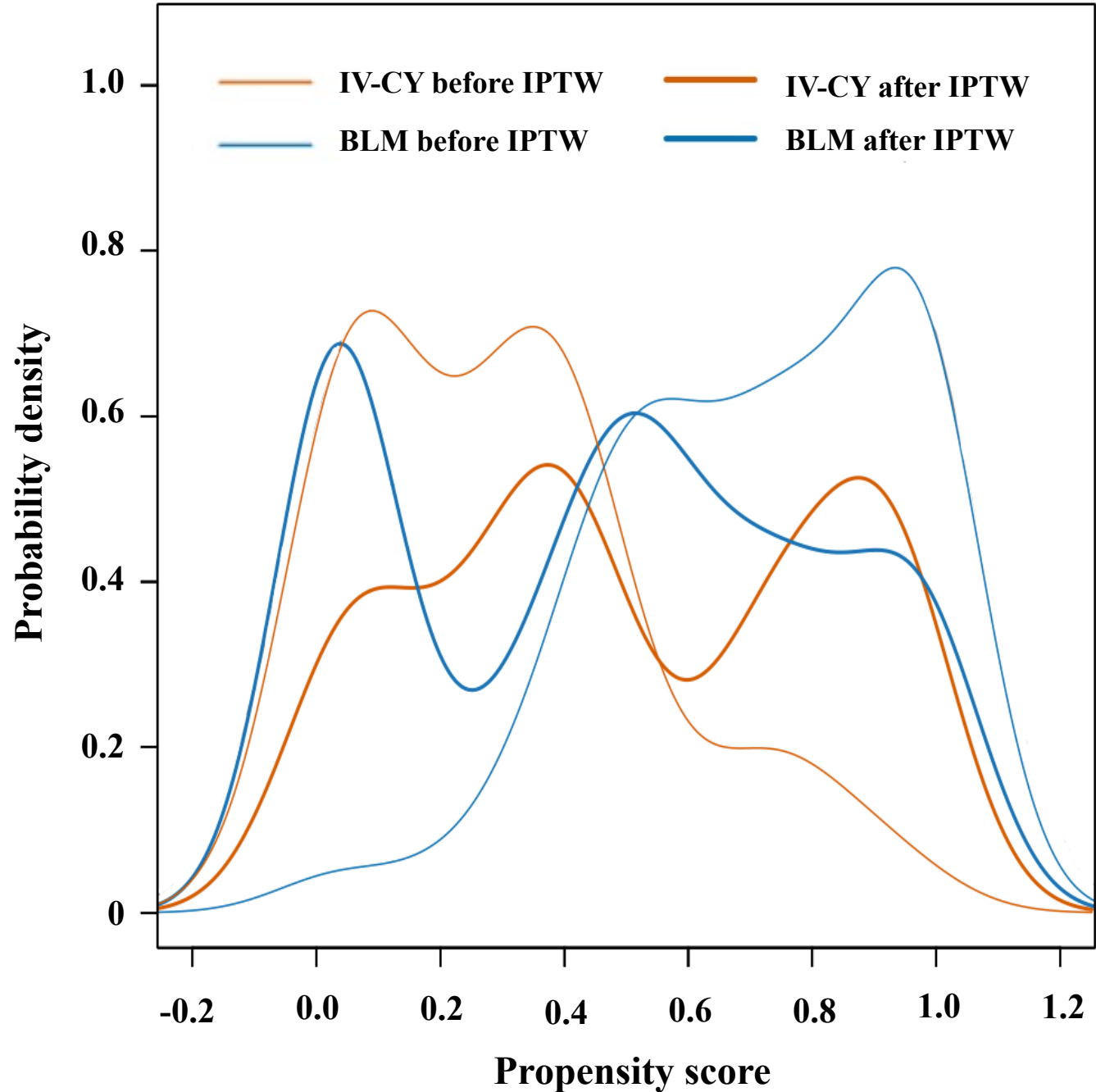

Supplement: Supplementary file 1 [file jcm-12-07627-s001.zip › jcm-2693915-supplementary.pdf]
